# Fixation duration and the learning process: an eye tracking study with subtitled videos

**DOI:** 10.16910/jemr.13.6.1

**Published:** 2020-08-16

**Authors:** Shivsevak Negi, Ritayan Mitra

**Affiliations:** Indian Institute of Technology Bombay, Mumbai, India

**Keywords:** Learning process, Eye tracking, Fixation duration distribution, Multiple linear regression, Subtitled educational video

## Abstract

Learning is a complex phenomenon and education researchers are increasingly focussing on processes that go into it. Eye tracking has become an important tool in such research. In this paper, we focus on one of the most commonly used metrics in eye tracking, namely, fixation duration. Fixation duration has been used to study cognition and attention. However, fixation duration distributions are characteristically non-normal and heavily skewed to the right. Therefore, the use of a single average value, such as the mean fixation duration, to predict cognition and/or attention could be problematic. This is especially true in studies of complex constructs, such as learning, which are governed by both cognitive and affective processes. We collected eye tracking data from 51 students watching a 12 min long educational video with and without subtitles. The learning gain after watching the video was calculated with pre- and post-test scores. Several multiple linear regression models revealed a) fixation duration can explain a substantial fraction of variation in the pre-post data, which indicates its usefulness in the study of learning processes; b) the arithmetic mean of fixation durations, which is the most commonly reported eye tracking metric, may not be the optimal choice; and c) a phenomenological model of fixation durations where the number of fixations over different temporal ranges are used as inputs seemed to perform the best. The results and their implications for learning process research are discussed.

## Introduction

Research in education investigates learning through three distinct prisms. The first prism deals with what goes into learning, which includes the learning environment, learning content, and teacher and learner characteristics. The second prism involves the learning outcomes that are often based on summative features, such as grade point averages (GPA) or test scores. The third prism concerns itself with the learning processes that link the first and the third prisms (1). This area of research investigates the cognitive and affective mechanisms, strategies, and mental states that learners employ and how those might interact to produce the complex construct of learning. There is comparatively less research on learning processes but it has found a new lease of life with advanced computer hardware and psychophysiological sensor technologies such as eye tracking (2, 3), facial emotion recognition (4), EEG (5) and GSR (6). These technologies have the ability to track neurophysiological and behavioral parameters at an unprecedented temporal resolution, which can shed light into the minute by minute breakdown of the underlying processes in learning, such as cognition, affect, metacognition and motivation, or what is often referred to as the CAMM processes (7). What was earlier possible only through post-hoc surveys, interviews, human observations, and think-aloud protocols can now be achieved easily through non-intrusive computer technologies and that too at an unprecedented resolution.

Eye tracking has been particularly useful in the study of learning processes. For example, Liu (8) used total fixation duration, a commonly used eye tracking metric, to study learners’ reading processes when working with concept maps. The author found shorter total fixation duration near task-relevant areas when concept maps were used as a study aid implying easier comprehension of content. The scan-paths of fixations revealed concept map reading strategies. These results help us understand not only if concept mapping was useful as a study aid, which can be equally obvious from pre-post test scores in a between-subjects study design, but they also tell us how and why we observed differences in learning gain. Similarly, Mitra, McNeal, and Bondell (9) studied graphical problem solving using eye tracking data. They delineated problem-relevant graphical elements and used fixations to reveal strategic differences between high and low performers. As pupil diameters are correlated with cognitive load, the authors used this proxy relationship to differentiate between high and low performers based on their cognitive load demands.

### Role of average fixation duration in learning process research

Just and Carpenters’ influential eye-mind hypothesis proposed that there is no significant latency between what the eyes fixate on and what the mind processes (10). This set the stage for modern-day eye tracking research in the early 1980s. Ever since, average fixation duration has occupied a key space in eye tracking research (11, 12, 13). As much as this early body of work was situated in reading research the usefulness of fixation duration soon started percolating to other areas such as visual search, auditory language processing, mathematics, numerical proficiency, problem-solving, multiple representations, etc.(11). Between 1990 and 2000 approximately 1300 scientific articles had fixation duration in the title. That count has multiplied more than ten times between 2010 and the publication date of this article.

Rayner (11) in a comprehensive review of eye tracking research of the past twenty years, had suggested that eye movement data held great promise in information processing tasks but significant differences exist between task categories and it may not be prudent to generalize findings across categories. This caveat is easily verified through a meta-analytic study of expertise development in visual comprehension, which found high heterogeneity in effect sizes (14). Average fixation durations showed no clear pattern (indicating opposing directionality between studies) for either task complexity or visualization characteristics. In particular, the sum of evidence revealed that although experts indeed differed from novices in terms of having shorter fixation durations, they did not differ significantly from intermediates. More importantly and quite counter-intuitively, the intermediates seemed to have longer fixation durations than the novices.

The idea that average fixation duration should have some bearing on the processing demands of a task is almost intuitive. More complex tasks should require more processing time and hence it is expected that subjects may need to look longer to process all the information provided by the stimuli. Likewise, with growing expertise this processing demand relaxes because the expert can pull schemata relevant to the task from long-term memory, thereby reducing processing (9). A much less appreciated aspect of average fixation durations is whether the underlying construct of average fixation duration is stable across contexts. For example, if the task is a short duration visual search where participants need to quickly locate a target among distractors then the underlying construct that mediates average fixation duration could be attention, alertness, focus, concentration or some similar construct (15). However, if the task changes to attending an online lecture for a long duration (several minutes to an hour) and the goal is comprehension (as opposed to search) then fixations could represent a broader range of underlying constructs that they are responsive to. If we simply use average fixation duration to indicate learning then it would be a mistake because fixation durations are not likely to be influenced by some ‘super-construct’ of learning. Instead, we argue, fixation durations could be influenced by latent affective processes that lead to learning. For example, a learner could be distracted, bored, confused or engaged, at different times during the lecture, which would influence his learning and should, hypothetically, leave their imprint on fixation durations as well. Given this complexity and temporal longevity of learning, the use of an average value for fixation durations as a proxy of learning would be a simplistic approximation, and possibly erroneous, in the context of educational research.

### Classification of fixation types

**Figure 1.**
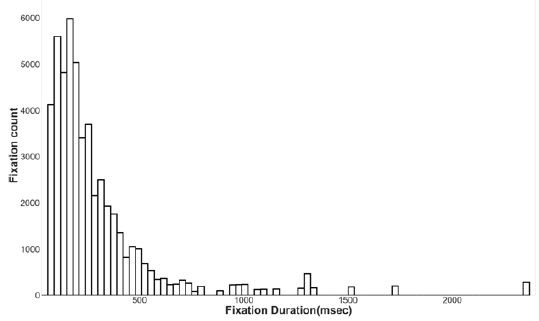
Representative frequency distribution of raw ﬁxation durations (n = 51238) from a single participant.

The distribution of fixation durations is quite predictable, famously non-normal and characteristically skewed to the left with typical median durations of 200-250 ms, mean durations of 300-350 ms, and an extended right tail of long and very long fixations (11, 16, 17) (see Fig. 1). In eye movement research fixations have been traditionally classified into ambient and focal types. Ambient visual fixations are short in duration and do not permit for conscious identification of visual objects. On the contrary, focal fixations are comparatively long in duration and reflect the conscious perception of objects. In spatial processing research, they seem to reflect either involuntary spatial processing (ambient) or conscious perception of visual objects (focal). Although the general definition and role of ambient and focal fixations are agreed upon their classification is not as straight-forward.

Velichkovsky et al. (18) originally characterized ﬁxations as ambient or focal based on their durations and the amplitude of the succeeding saccade. Unema et al.(16) further suggested that larger saccade amplitudes and shorter ﬁxation durations during the initial image viewing period represented ambient processing and that smaller saccade amplitudes and longer ﬁxation durations during the later viewing period represented focal processing. Providing further support for such classification between ambient and focal visual processing, Helmert et al. (19) found that viewers were more likely to remember objects when ﬁxation duration was long but the subsequent saccade amplitude was short, compared to when ﬁxation duration was short and the subsequent saccade amplitude was long. Krejtz et al. (20) used an ambient/focal attention coefficient, deﬁned as the relation between the current ﬁxation duration and the subsequent saccade amplitude to classify fixations. Krejtz et al. (21) formally defined the ambient/focal attention coefficient proposed by Krejtz et al. (20) as K, which acts as a dynamic indicator of ﬂuctuations between ambient and focal visual processing modes and permits statistical comparison between individuals and groups. They suggested negative values of coefficient K represent ambient processing and positive values represent focal processing. The interpretation of the null value of K remained ambiguous; it may mean long (short) ﬁxations followed by long (short) saccades. Although both seem to be rare, a better understanding of the underlying process is desirable yet currently unavailable. In another study, Holmqvist et al. (22) discussed a method of classification of focused (focal) versus overview (ambient) visual behavior. This technique was based more on transitions between areas of interest (AOI) rather than on the relation of ﬁxation durations and subsequent saccade amplitudes, and therefore, the findings are not easily generalizable to studies that did not look at transitional analysis of eye movement data. In the aforementioned studies, it seems that classification was primarily based on saccade amplitude with relatively less reliance on exact fixation durations. Follet et al. (23) even claimed that classification of visual fixations can be achieved using saccade amplitude alone and discounted the role of fixation duration in such classification (Table 1).

A different approach in ambient-focal classification is based only on fixation durations without considering saccade characteristics at all (24). Such classification suggests short fixations (50-150 ms) to be of ambient type while long fixations around 300-500 ms are considered to be focal (17). These two populations of fixations, ambient and focal, with small differences in the actual ranges used by researchers (see Table 1), have often been reported in eye-movement research. It may be noted, however, the fixation durations for the corresponding categories are different across the two classification schemes that we discussed. For example, a common range in the fixation only classification scheme happens to be around 300-500 ms, which is greater than the values of focal fixations suggested in the classification based on fixation and saccade amplitude (Table 1).

**Table 1.**
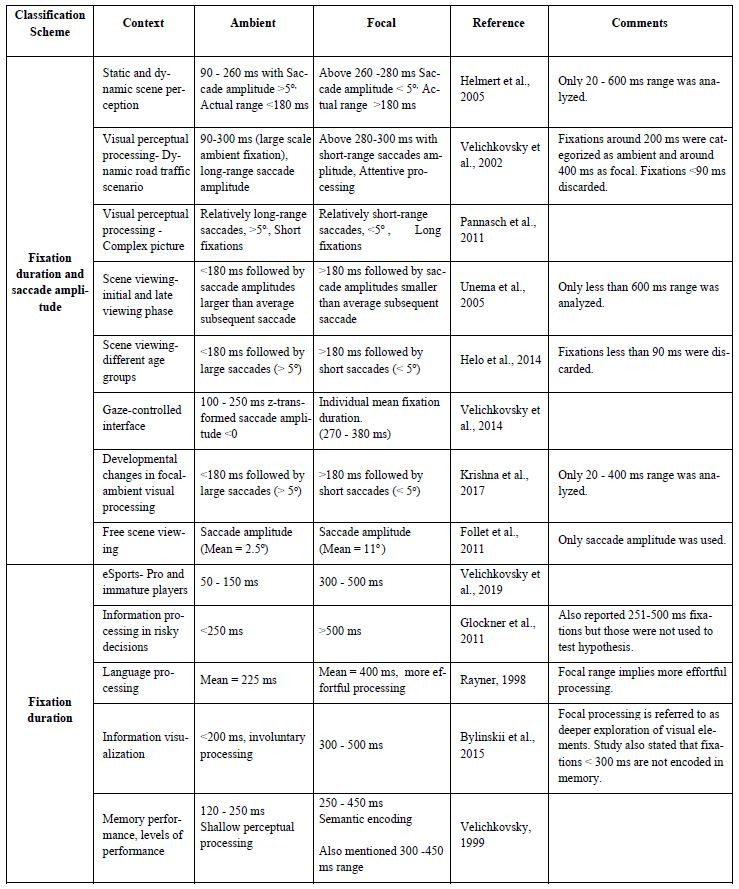
Summary of literature review based on ambient and focal fixation classification.

The fixation durations in an eye tracking dataset vary from as small as 50 ms to several seconds (over 2000 ms (17, 31). The aforementioned ambient/focal classification of fixations does not adequately represent this wide range. Indeed, as indicated in Table 1, several authors have not used the full range of fixation durations and have restricted their analyses to a few hundreds of milliseconds. Such decisions are possibly justified based on the type of tasks that have accompanied such studies as explained in the Introduction. However, in case of learning tasks, restricting our analysis to such a narrow range of fixation durations could be fraught with errors as very long fixations could indicate negative academic affects such as confusion, boredom, and frustration. Some empirical results suggest very long fixation durations to be indicative of negative affect such as those related to confusion (32, 33, 34) or cognitive load (35), although a potential confound with interest cannot be ruled out (36).

## Research Questions

In this study we look at fixation duration distributions and their usefulness in the study of learning processes. As outlined in the Introduction and Table 1, our understanding of fixation duration is largely from studies outside the context of learning and can, therefore, pose a challenge. Hence, evaluation of the role of fixation duration in learning process research is quite timely and useful. Fixation durations are typically right-skewed with a long tail and previous workers have outlined the heterogeneity of fixation vis-a-vis the type of cognitive processing that they represent. In view of such evidence, we think it is apt to re-evaluate the role of average fixation duration in learning process research. The use of a mean fixation duration may be unjustified both from a geometric perspective as well as a process perspective. A skewed distribution is unlikely to be well-represented by a single mean value of the fixation durations and studies that have used this common metric (37, 38, 39 ,40, 41, 42) could have extracted useful insights had they incorporated fixation distribution characteristics in their analysis. This concern is purely geometric in nature and is solely dependent on the shape of the distribution regardless of the cause behind such a distribution. More importantly, in a context as complex and lengthy as learning, a single mean may be a conceptually unsound choice as well because of the underlying affective controls of learning and how those affective states influence fixation durations. Specifically, for the purpose of this study, we have the following research questions.

RQ1: Is there empirical evidence to justify the use of average fixation duration for research on learning processes?

RQ2: Is average fixation duration a *necessary* and *sufficient* measure to study learning processes?

RQ3: Is there an alternative model for the representation of learning processes with fixation durations?

## Methods

### Participants

A total of 51 male participants (16-18 years old with no vision problem) from a polytechnic institute in India voluntarily participated in the study. The exclusively male population was not because of selection bias as enrolled students were exclusively male as well. The goal of polytechnic institutes in India, as elsewhere, is to make students employable for entry-level industry jobs by imparting industrial training using an apprenticeship model of learning. This institute in particular is geared toward supplying the lowest rung of employment such as for jobs as an electrician, automobile mechanic, plumber, refrigerator technician, etc. As a result, a large majority of the student population are from socio-economically backward strata of the society with an average annual income of less than 4600 USD. The participants had very poor English comprehension skills and everyone spoke the same native language, Marathi. Though the medium of instruction in the institute was English (L2) the rudimentary English language skill, both verbal and written, necessitated any conversations with the students to be conducted primarily in the Marathi language. The poor financial condition compels them to leave mainstream education such as 4 years engineering/medical/commerce degree programs, and join such short term diploma courses (one to two years in duration) that impart job-ready industrial skills. The minimum qualification of enrolment was passing of the 10^th^ grade examination.

The study was approved by the local institutional ethics committee (No.IITB-IEC/2019/012) and all participants provided informed written consent.

### Materials

The materials used in this study included a demographic data sheet, educational video, a pre-test, and a post-test. The demographic questions pertained to age, enrolled courses, last class attended, family income, etc. In consultation with the course instructor, a 12 minute long educational video introducing the basic concept and architecture of the World Wide Web and the internet was chosen. The voice-over was in Indian English and the video lecture contained presentation slides with textual and graphical information. The VLC player was used to embed either English or Marathi subtitles. A maximum of two lines were used to display subtitles on the screen. Before eye tracking data collection participants’ prior knowledge about the topic was assessed with the pre-test comprising 14 multiple choice questions, which were designed in consultation with the course instructor. Similarly, 14 multiple choice questions, different from the pre-test questions but of similar difficulty level, were used to formulate the post-test questionnaire. The pre- and post-test questionnaires were drafted in English and language experts translated all questions to Marathi so that the students could have both versions, English and Marathi, when answering the questions. Both the tests were untimed.

### Procedure

The participants were instructed to watch the video lecture after completing the demographic survey and the pre-test questionnaire. A screen-based Tobii eye tracker (X3-120) operating at 120 Hz recorded the eye movements of the participants as they watched the video lecture. The stimulus was displayed on a 17- inch screen and participants were seated comfortably in a sufficiently illuminated room on a stable chair at a distance of approximately 680 mm from the stimulus screen. The minimum fixation duration that can be recorded by the eye tracker was 66 millisecond. A five-point calibration procedure was used to calibrate each participant’s eyes before the experiment. During the recording session, participants were not allowed to use the mouse and keyboard or pause video. Tobii Pro Lab (version 1.123) was the software used for data collection and analysis. A post-test was administered immediately after the participants completed watching the video lecture.

The video was watched with Marathi subtitles (MS) by 19 participants, with English subtitles (ES) by 16 participants and with no subtitles (NS) by 16 participants. One goal of our study was investigating the impact of subtitles within educational context hence a between-subjects study design was chosen (43, 44). We will report group differences of self-reported measures, performance and eye tracking measures separately. In this article, however, we choose not to focus on group differences. All statistical analyses were conducted with Jamovi (Version 0.9.5.13), an open-source software project (45).

## Results

### Learning gain

**Figure 2.**
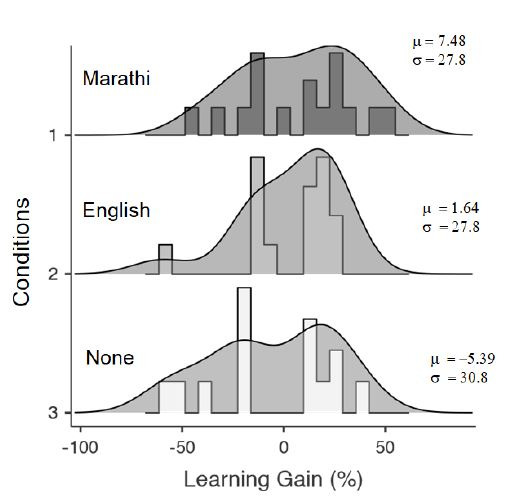
Histograms representing distributions of Hake’s learning gain calculated for each participant under three different conditions (Marathi subtitle, English subtitle and No subtitle). The group means and standard deviations are reported.

A total of 6 participants (3 from MS, 1 from ES and 2 from NS) were rejected at the onset of analysis due to very low eye gaze data collection. Learning gain was computed using Hake’s formula given by (S^post^ – S^pre^) / (S^max^ – S^pre^), where S^post^ represents the post-test score, S^pre^ represents pre-test score and S^max^ represents the total score for the test (46). The learning gains of 2 participants in the ES group and 1 participant in the NS group were more than 2 SD (SD=28.7) away from the combined group (MS+ES+NS) mean (M=0.439) and excluded from further analysis (Fig. 2). One way ANOVA of learning gains between the three groups was insignificant [F (2, 39) = 0.524, p = 0.596].

### Eye Movement Analysis

**Figure 3.**
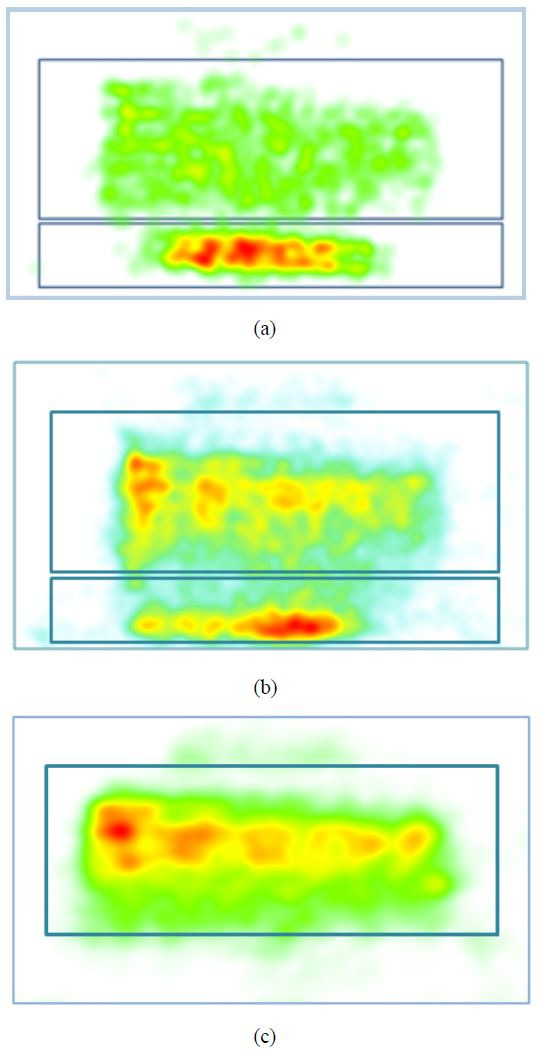
Heat maps for (a) MS, (b) ES, and (c) NS groups. It is evident that MS and ES groups paid attention to the subtitle area apart from attending to the content area (more red/dark shades). Red/dark shade indicates high density of fixations and green/light shade indicates low density. The two analytical sections are the Content_AOI_ (upper box) and Subtitle_AOI_ (lower box, when present).

Two areas of interest (AOIs) were identified based on heat maps (Fig. 3), namely, content (Content_AOI_) and subtitle (Subtitle_AOI_). The content area displayed the main learning content; however, such nomenclature does not imply subtitles do not provide any learning content. The descriptive statistics of fixations recorded for the three groups are provided in Table 2. The pooled fixation durations from the MS and ES groups were used for the multiple regression analysis (N=29). The data from the NS group (N=13) were analyzed separately (see Discussion). The reason for clubbing the two groups with subtitles for analysis stems from a learning process perspective. Subtitles generate a lot of attention as evident from the heat maps of fixations (Fig. 3a, 3b). The conscious act of reading a subtitle or not can influence understanding of the content. Although the subtitle language can influence such comprehension, as suggested by the difference in fixation density in Figs. 3a and 3b (Subtitle_AOI_), the process of sense-making from subtitled videos is expected to remain similar across language groups, as revealed in the similarities between Fig. 3a and 3b. The same cannot be said about videos without any subtitling (Fig. 3c). Furthermore, from an analytical perspective, this grouping ensures parity between NS and the other two groups as the areas of interest, Content_AOI_ and Subtitle_AOI_, are relevant only for the MS and ES groups but not for the NS group.

**Table 2.**
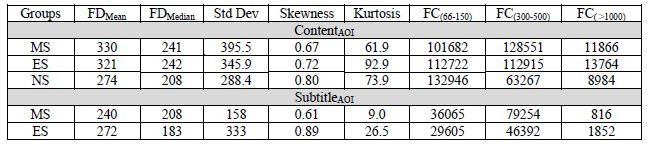
Descriptive statistics for the three groups (MS - Marathi subtitle; ES - English subtitle and NS - No subtitle) and two areas of interest (AOI) – content and subtitle. FD stands for fixation duration in milliseconds. FC stands for fixation counts and the subscripts refer to the range in milliseconds. Kurtosis and Pearson’s coefficient of skewness are also reported.

Multiple linear regression analyses were conducted to model the variability in post-test scores caused by different subsets of independent variables, namely, the pre-test scores, the mean of fixation durations, the median of fixation durations, and the three distinct non-continuous and non-overlapping fixation duration categories that are based on prior work (see Introduction and Table 1), after controlling for subtitle language. The number of fixations less than 150 ms (but greater than 66 ms), F_150, loosely correspond to the ambient fixations of prior work. Similarly, the number of fixations between 300 and 500 ms, F_300-500, represents focal fixations, and the number of fixations greater than 1000 ms, F_1000, includes fixations that may be considered to be too long. The results from the multiple linear regression models are provided in Table 3. For all the models, assumptions of autocorrelation and normality of residuals were not violated and corresponding test results remained within reasonable values. Some specific cases of multicollinearity are discussed in the appropriate context below.

**Table 3.**
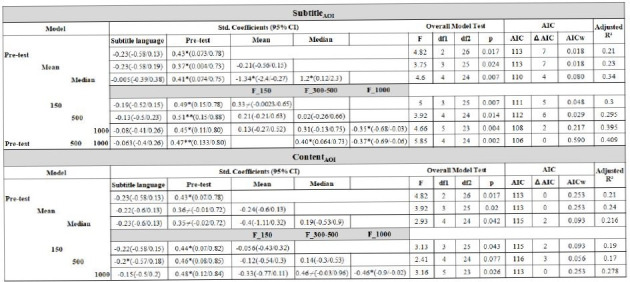
Results of multiple linear regression models. independent variables (IVs) are listed in the far left columne. Each model has all the IVs from the one above exept in the last model of Subtitle AOI where all the IVs are listed. Significance levels < 0.01, < 0.05 and < 0.1 are marked as **, * and /, respectively.

### Subtitle

For the preliminary model we use only pre-test scores, which expectedly predicts post-test scores and explains 21% of the variation (F=4.82, p= 0.017, AIC_w_ =0.018 , Adjusted R^2^ = 0.21). On adding mean as a predictor the model remains comparable (F= 3.75, p= 0.024, AIC_w_ =0.018, Adjusted R^2^ = 0.23). Upon adding median to the list of predictors there is a substantial increase in predictability to 34% (F=4.6, p= 0.007, AIC_w_ =0.08, Adjusted R^2^ = 0.34). We do note that the obvious concern of multicollinearity is valid in this case as revealed by the high VIF (VIF=11.87). However, contrary to popular belief “the fact that some or all predictor variables are correlated among themselves does not, in general, inhibit our ability to obtain a good fit nor does it tend to affect infer-rences about mean responses or predictions of new observations” (47), although it does affect the coefficients and p-values of the predictors that show multicollinearity.

Excluding both the measures of central tendency, only pre-test scores and F_150 provide a reasonably good fit with an adjusted R^2^ value of 0.3 (F=5.0, p= 0.007, AIC_w_ =0.048 ). Adding F_300-500 to the model does not yield any substantially different model fit (F=3.92, p= 0.014, AIC_w_ =0.029, Adjusted R^2^ = 0.295). However, adding F_1000 clearly improves the model to a predictability of 39.5% (F=4.66, p= 0.004, AIC_w_ =0.217, Adjusted R^2^ = 0.395). A more parsimonious model with marginally better adjusted R^2^ value and lowest AIC is obtained by excluding F_150 from this model (F=5.85, p= 0.002, AIC_w_ =0.590, Adjusted R^2^ = 0.409). This could indicate some collinearity issues between F_150 and F_300-500 although the VIF measure were reasonable in this case (F_150, Tolerance = 0.6, VIF = 1.67; F_300-500, Tolerance = 0.88, VIF = 1.14; Pearson’s r = 0.61).

### Content

The model for pre-test remains the same for both categories. However, the model does not appreciably improve upon adding mean (F=3.92, p= 0.02, AIC_w_ =0.253, Adjusted R^2^ = 0.24) and median (F=2.93, p= 0.042, AIC_w_ =0.093, Adjusted R^2^ = 0.216) although the model with pre-test and mean performs the best out of these three models. Without the central tendency measures and adding only F_150 to pre-test scores makes for a worse fit (F=3.13, p= 0.043, AIC_w_ =0.093, Adjusted R^2^ = 0.19). Adding F_300-500 does not improve the model fit (F=2.41, p= 0.077, AIC_w_ =0.056, Adjusted R^2^ = 0.17). Adding F_1000 improves the model substantially with a predictability of 27.8% (F=3.16, p= 0.026, AIC_w_ =0.253, Adjusted R^2^ = 0.278).

## Discussion

RQ1: Is there empirical evidence to justify the use of fixation durations for research on learning processes?

In all the multiple linear regression models pre-test scores alone have a low yet consistent explanatory power of the variability observed in the post-test scores. This is only to be expected because pre-test scores test prior knowledge, which is an important indicator of performance in post-tests. If fixations had not played any role in the underlying learning process then the addition of such predictors should not have improved the predictive power of the models. However, most of our subsequent models that used at least one measure of fixation duration (either a central tendency measure or a fixation duration count measure) explain more of the variability in the learning gain compared to the model with only pre-test scores. This indicates that something fundamentally linked to performance in the tests was being captured by the fixation measures and learning would be the most likely candidate for that.

The model results provide further insight. The models for the subtitle section with a highest adjusted R^2^ value of 0.409 clearly outperform the models of the content section with a maximum adjusted R^2^ value of 0.278. In other words, fixation duration should have higher explanatory power when subtitles were included. Heat maps of fixations for the MS and ES groups also indicate the relative importance participants placed on these two sections (Fig. 3a, 3b). Indeed, if no subtitles were provided, then fixation durations should have low explanatory power and the overall model fit should deteriorate as learning can be expected to suffer without any form of subtitling.

In this context, it would be useful to check how such a model performs with the NS group data (N=13) where there was no subtitling. As expected, the model predictability was low for any combination of predictors, including pre-test scores only. The best model was with pre-test scores alone (F=2.60, p= 0.135, Adjusted R^2^ = 0.118). By adding fixation based predictors the model fit worsened. Thus, neither the pre-test score nor any of the fixation measures, or any of the combinations thereof, were able to predict the variability observed in the post-test scores with reasonable success. This is consistent with our expectation that without any subtitling this group would remain confused about the material being taught and the poor performance could be symptomatic of either poor comprehension or random test-taking.

We also need to ask a related question; can we quantify the learning that takes place? A definitive answer to this question is outside the scope of this work but we do want to highlight the fact that the results open up interesting possibilities for using fixation duration as a *process metric*. In this operationalization of the term, a process metric is a small step ahead of a *process measure,* which we define as simply a tool or a set of tools to understand the learning process. For example, scan paths have been used as a tool to reveal the process of learning (35) and can be considered as a process measure. However, for it to be considered as a metric the variability of scan paths across tasks and user types need to be quantified. This would be a challenging task given the exploratory nature of scan paths and the inherent qualitative nature of interpretation that usually accompanies it. In our opinion, something that is easily quantifiable such as fixation duration would be more suitable as a learning process metric. These results suggest such a possibility but before we arrive at such a metric generalizability of these results need to be tested with a variety of learning media and contexts.

RQ2: Is the mean fixation duration a necessary and sufficient measure to study learning processes?

Several studies have used the arithmetic mean of fixation durations. As discussed earlier the fixation duration distribution is typically right-skewed and it has been suggested that the mean may not be capturing the fixation duration distribution adequately. In RQ2, we have proposed to test this premise by asking whether the mean is a necessary and/or a sufficient measure of fixation durations.

The best model for the subtitle section (Adjusted R^2^ = 0.409) as well as the content section (Adjusted R^2^ = 0.278) does not require the mean which suggests that mean is not a necessary measure. Also, we do see a marked improvement in model fit when the median is used with the mean (Adjusted R^2^ = 0.23 increases to 0.34). This suggests that simply using the mean may not sufficiently capture the variability in test performance.

This finding also flags a more subtle concern in using a mean value for fixation durations and one that we have discussed earlier in the Introduction. The fact that even a combination of mean and median can fall short of explaining the variability observed in the dependent variable indicates that the problem probably rests on what those values truly represent than the geometric nature of the distribution. If the usage of the mean of fixation durations suffered only because of the underlying non-normality and/or skewness then addition of a median would have invariably improved the model fit. However, the fact that this is not seen in the data indicates a different underlying cause. Averages of any kind, be it mean or median, assumes the values being averaged to be of one kind. If learning were a unidimensional construct and the fixation durations reflected that construct and only that construct alone, then averaging would have, at least, had some conceptual underpinning. However, several emotions regulate our learning and each of these emotions may have different fixation duration signatures. In that case, using an average measure is more than mathematically unsound; it is conceptually unsound as well. In the following section, we propose an alternative model to better reflect the fixation duration distribution within a learning context.

RQ3: Is there an alternative model for the representation of learning processes with fixation durations?

From the results discussed so far, we propose a phenomenological tripartite affective model of fixation durations. In this model, very small fixations (less than ~F_150) represent negative valence (not focusing, being distracted, etc.), medium duration fixations (~F_300-500) represent positive valence (such as, focused attention) that is beneficial to learning and very long durations of fixation (~F_1000) represent negative valence (e.g., zoning out or confused) that is not beneficial to learning. This tripartite model is loosely in keeping with the observations from previous studies that were presented in the Introduction. We, however, refrain from pinning down the exact emotions vis-a-vis their fixation duration signatures and restrict ourselves to positive valence/beneficial to learning and negative valence/detrimental to learning classification only. Based on our empirical results if such a model is found to be valid we would argue the explanatory power of the measures of central tendency is only as much as those measures are tied to the most task-relevant fixation category. For example, the mean may have a greater predictive power in a learning scenario if and only if the mean value represents the category (ies) of fixation; namely, F_150, F_300-500, and F_1000, that was (were) most relevant to the task and/or user. Likewise, mean and median together would have greater predictive power if the two categories best represented by these values were relevant for the task and/or user.

**Figure 4.**
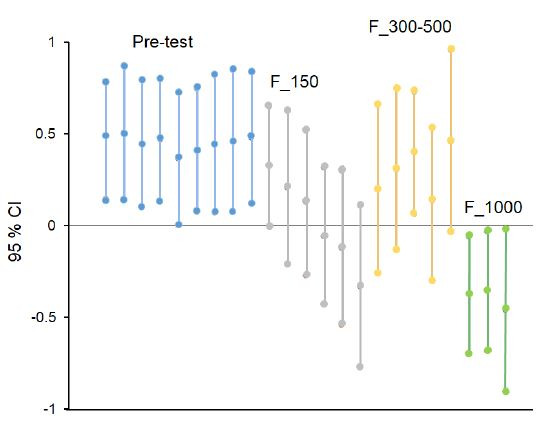
An ensemble of confidence intervals (95%) from the regression models listed in Table 3. The labels align with the first CI of that category. Light blue, grey, yellow and green color bars represent CIs for the pre-test, < 150 ms fixation duration, 300-500 ms fixation duration and > 1000 ms fixation duration, respectively.

We now use empirical evidence from this study to validate such a tripartite model. Fig. 4 demonstrates an ensemble of confidence intervals (95% CI) for the standardized coefficients of the various models outlined in Table 3. The pre-test score coefficients have 95% CIs that are greater than zero, which indicates, as expected, an unambiguous positive correlation between pre- and post-test scores.

All 95% CIs for F_1000 are less than zero indicating that as supposed in our model very long fixations (lasting for more than one second) are indeed detrimental for learning. Therefore, we can be sure, at the very least we have a bipartite model of fixation. Evidence for a tripartite model, where very small fixations are also detrimental and a middle range of fixations, also referred to as focal fixations in some contexts, that is beneficial to learning, is more nuanced. As expected from the model, most 95% CIs of F_300-500 are greater than zero, which indicates these fixations are indeed positively correlated with post-test scores. Additionally, the effect sizes (standardized coefficients) of the pre-test and the F_300-500 fixations are similar indicating equal contribution to learning outcomes. This result supports our proposed model as the F_300-500 fixations seem to be contributing positively and adequately toward learning.

However, for the proposed tripartite model to work the F_150 fixations need to be negatively correlated with the learning outcome. The interpretation of F_150 is less straightforward as the 95% CIs traverse both negative and positive territories in Fig. 4 and also overlap with F_300-500. Therefore, it seems that the valence (positive vs. negative academic emotion) that was well differentiated at the upper end of the fixation duration range (between F_300-500 and F_1000) is less so at the lower end.

There could be several reasons for the above results. First and foremost, the tripartite model may not be accurate. However, there is a marked dip in the 95% CIs of F_150 fixation durations into negative territory, which is not the case for F_300-500. Likewise, we see the possibility of the F_300-500 coefficients to be also becoming negative although demonstrably they are mostly in positive territory. These two facts suggest a more likely alternative. The differentiation between productive and non-productive fixations at the low end of the spectrum is likely more fuzzy and context-dependent. For example, from the literature we know that very short fixations can suggest expertise; a proficient reader does not need to fixate for long durations (14, 48). However, small duration fixations may also suggest distraction as we had originally proposed. Hence, instead of outright rejection of a tripartite phenomenological model, we believe we have evidence for a softer version of this model. In such a model, very long fixations are detrimental, which is followed by a beneficial range of fixations whose lower limit can extend to very small fixations and one which could be dependent on task/learner characteristics. Only after reaching this limit the fixations may contribute negatively toward learning. Likewise, the range of detrimental fixations could well expand into the 300-500 ms range, which again would again be sensitive to the context.

## Limitations

1) This study only explores a specific learning context. If we attach too much significance to this study alone, we risk making the same mistakes as the studies that do not take into consideration task and domain differences. More studies with a variety of contexts are required to generalize the proposed model.
2) The boundaries of fixation durations were drawn loosely from literature and based on fixations alone. However, we do not make any claim for the specific numbers to be invariable. Instead, the goal of the article was to suggest a plausible affect-based fixation duration model that can explain variation in test scores.
3) Finally, we lacked evidence to suggest a truly tripartite model and the boundary at the lower end (between F_150 and F_300-500) seemed to be fuzzy. However, from this one study we cannot also be sure whether the boundary at the upper end (between F_300-500 and F_1000) is as clear-cut as it seems, especially because, in some learning contexts, distraction and interest are likely to be two contrasting affect that can influence such long fixation durations.
4) The analysis solely employs fixation duration ranges. As outlined in the Introduction, both saccade and fixation have been used to classify fixations into ambient and focal types. Therefore, it is quite conceivable that classifying fixation with information from saccadic eye movement would improve the model results.

## Conclusion

The findings revealed that fixation duration can be a useful metric to trace the learning process. Within the context of this study, central tendency measures of fixation durations could not adequately explain the variability observed in the post-test scores. A tripartite phenomenological model based on affective processes that underlie learning was proposed. In this model, short or ambient fixations (<150 ms) and very long fixations (>1000ms), contribute negatively to learning, whereas focal fixations (300-500 ms) contribute positively. So far we have been able to validate a softer version of this model where the demarcation of useful from useless fixation at the lower end of the spectrum is fuzzy. One possible reason for the ambiguity in the classification of short fixations into either detrimental or beneficial for learning could be the observed effect of expertise on fixation durations. As experts tend to require shorter fixations than novices, the F_150 range is possibly representing expertise as well as a negative learning affect.

### Ethics and Conflict of Interest

The authors declare that the contents of the article are in agreement with the ethics described in http://biblio.unibe.ch/portale/elibrary/BOP/jemr/ethics.html and that there is no conflict of interest regarding the publication of this paper.

### Acknowledgements

The authors are grateful to four anonymous reviewers for their comments, which greatly helped in improving the manuscript. This work was supported by Seed and IRITPP funds provided to Ritayan Mitra by the Indian Institute of Technology Bombay.
